# CSF p-Tau levels in the prediction of Alzheimer's disease

**DOI:** 10.1242/bio.20135447

**Published:** 2013-09-04

**Authors:** Ramesh J. L. Kandimalla, Sudesh Prabhakar, Willayat Yousuf Wani, Alka Kaushal, Nidhi Gupta, Deep Raj Sharma, V. K. Grover, Neerja Bhardwaj, Kajal Jain, Kiran Dip Gill

**Affiliations:** 1 Department of Biochemistry, Post Graduate Institute of Medical Education and Research, Chandigarh, India 160 012; 2 Department of Neurology, Post Graduate Institute of Medical Education and Research, Chandigarh, India 160 012; 3 Department of Anaesthesia and Intensive Care, Post Graduate Institute of Medical Education and Research, Chandigarh, India 160 012

**Keywords:** p-Tau, North Indian patients, Sensitivity, Alzheimer's disease

## Abstract

The two hallmarks of Alzheimer's disease (AD) are neurofibrillary tangles and amyloid plaques. Neurofibrillary tangles are formed due to the hyperphosphorylation of tau protein. There is an urgent need to develop a reliable biomarker for the diagnosis of AD. Cerebrospinal fluid (CSF) is surrounding the brain and reflects the major neuropathological features in the AD brain. Diagnosis, disease progression and drug actions rely on the AD biomarkers. Mainly CSF tau and phosphorylated tau (p-Tau) have been observed to serve the purpose for early AD. Keeping in view the early appearance of p-Tau in CSF, we analyzed p-Tau levels in 23 AD, 23 Non AD type dementia (NAD), 23 Neurological control (NC) and 23 Healthy control (HC) North Indian patients. The levels of p-Tau were found to be increased in AD patients (67.87±18.05 pg/ml, SEM 3.76) compared with NAD (47.55±7.85 pg/ml, SEM 1.64), NC (34.42±4.51 pg/ml, SEM 0.94) and HC (27.09±7.18 pg/ml, SEM 1.50). The resulting sensitivity for AD with NAD was 80.27% whereas with respect to the NAD, NC and HC was 85.40%. Therefore elevated levels of p-Tau in AD can be exploited as a predictive biomarker in North Indian AD patients.

## Introduction

Alzheimer's disease (AD) is a neurodegenerative disorder characterized by a slowly progressive dementia and brain atrophy. The window period for the AD may be around 15 years before the onset of clinical symptoms. In the window period the disease process and continues but both patient and clinician remain ignorant (Kandimalla et al., 2011a; Kandimalla et al., 2011b). In western society and developing countries with the increasing life expectancy AD becomes one of the health problems in the elderly population. For promising therapeutics, it requires differential biomarker for the early detection of AD. Based on the Alois Alzheimer observations, there are differences in the dyed brain fibrils of a patient afflicted with dementia compared with fibrils in a normal brain. Thus, it is generally accepted that there are differences in the composition of neurofibrils. Accumulation neurofibrillary tangles (NFTs) intraneuronally are one of the pathological hall marks of AD (Lewczuk et al., 2004; Kandimalla et al., 2012; Blennow et al., 2010). In more than 80% of AD patients the most consistent finding protein is microtubule associated protein i.e. tau in cerebrospinal fluid (CSF) which has direct contact with brain, where the change/modification in the protein levels can be monitored biochemically (Lewczuk et al., 2004). In AD brain tau protein accumulates in the form of hyperphosphorylated form. The determination of phosphorylated form of tau (p-Tau) may increase the specificity and sensitivity in the detection of AD in CSF as opposed to total tau (Blennow et al., 2010).

According to the current NINCDS-ADRDA criteria, for any candidate biomarker to ensure accuracy, it should be tested in patients with a diagnosis of Probable AD and the values obtained from AD patients should be clearly distinct from those of control subjects. Currently ‘tau’ is the most investigated marker in the diagnosis of AD. In normal brain tau is responsible for the assembly and stability of the microtubules in the axons of neurons and in turn responsible for the neuronal plasticity. In human brain, tau exists in six different isoforms with alternatively spliced exons. The largest tau isoform has 441 residues (tau441) and four repeat domains with exon 10 spliced in and two extra domains from exons 2 and 3 whereas smallest contains 352 amino acid residues and three repeat microtubule-binding domains (Blennow et al., 2010).

Vandermeeren et al. first showed increased CSF tau concentration in AD (Vandermeeren et al., 1993). Since then many studies have confirmed the increase of total CSF tau protein concentration in AD patients compared to controls (Kandimalla et al., 2011a; Kandimalla et al., 2011b; Tapiola et al., 2009; Haense et al., 2008; Hampel et al., 2003; Andreasen et al., 2003). In AD brain, p-Tau is the main component of paired helical filaments (PHFs), which form NFTs and involves in the formation of senile plaques extracellular. Therefore, CSF p-Tau levels may be considered as potential biochemical marker for AD instead of CSF total tau levels, because it represents directly to the neuronal (axon) degeneration. Several ELISA methods have been developed so far for the detection of different epitopes i.e. serine 235, threonine 231, threonine 181+231, serine 235 + threonine 231, serine 199 and serine 396+404 of p-Tau (Blennow et al., 2010). Andreasen in 2003 stated that several studies have shown CSF p-Tau levels are higher in AD patients when compared with healthy controls with a specificity of 92% and sensitivity of 80% (Andreasen et al., 2003). Previous studies revealed that with the p-Tau estimation, AD could be differentiated from other dementias such as front temporal dementia (FTD) and Lewy body disease with dementia (DLB) (Blennow et al., 2010). So, in this study, we evaluated the clinical usefulness of p-Tau as a biological marker in the diagnosis of AD in comparison with Non-AD type (NAD) dementia, Neurological controls (NC) and healthy controls (HC) initially in 23 patients in each group in North Indian population.

## Materials and Methods

After approval of the local Ethics Committee, informed consent was obtained from each patient or their caregivers. Altogether 92 patients were enrolled over 3 years in four groups.

### Alzheimer's disease (AD)

#### Inclusion criteria

Twenty three (23) AD patients, of either sex, above 50 years of age were recruited according to the NINCDS-ADRDA criteria (McKhann et al., 1984), International Classification of Diseases (ICD-10). Patients underwent extensive clinical neurologic examination including neuropsychological tests, Mini-Mental State Examination (MMSE), PGI Memory Scale, EEG, brain CT or MRI, routine laboratory tests, and CSF analysis.

#### Exclusion criteria

The patients who were less than 50 years with AD, doubtful AD, AD overlapping with other dementias and with other neurological diseases were excluded from this group.

### Non AD or other dementias (NAD)

#### Inclusion criteria

Twenty three (23) NAD patients, of either sex, more than 50 years, with dementias other than AD, like Front temporal Dementia (FTD), Parkinson's disease with Dementia (PDD), Hydrocephalus Normal Pressure with Dementia (HCNPD), Vascular Dementia (VD), Creutzfeldt–Jakob disease (CJD) and Un Classified Dementia (UCD). Patients were recruited according to the DSM-IV. Patients extensive clinical neurologic examination including neuropsychological tests, MMSE , PGI Memory Scale, EEG, brain CT or MRI, routine laboratory tests, and CSF analysis.

#### Exclusion criteria

The patients who were less than 50 years and with other neurological diseases were excluded in this group.

### Neurological controls (NC)

#### Inclusion criteria

Twenty three (23) NC group of either sex, above 50 years, with other neurological diseases for which dementia is not a component like polyneuropathy, motor neuron disease (MND), demyelination, multiple sclerosis, amyotrophic lateral sclerosis (ALS), Meningitis, encephalopathy, cervical myelopathy, sensory ataxia.

#### Exclusion criteria

Neurological diseases with dementia as a component, hyperthyroidism, cerebral palsy, psychomotor patients were excluded from this group.

### Healthy controls (HC)

#### Inclusion criteria

Twenty three (23) HC patients, of either sex, above 50 years were recruited from Orthopaedics and Urology department, Nehru Hospital, PGIMER, Chandigarh, where the patients were undergoing surgery and were cognitively normal.

#### Exclusion criteria

Patients with Diabetes, doubtful neurological disease and age less than 50 years were excluded from this group. AD, NAD, NC patients were recruited from Neurology Department, PGIMER, Chandigarh.

### CSF collection

CSF samples were obtained using a standardized protocol. Lumbar punctures were performed in mornings after an overnight fast at L3/L4 or L4/L5 interspaced. The first 2–3 ml of CSF was analysed for protein and glucose and then 1 ml aliquot was immediately frozen and stored at −80°C until biochemical assays for p-Tau levels was performed. Estimation of CSF p-Tau by ELISA: CSF levels of p-Tau phosphorylated at threonine-181 were measured by ELISA, using a commercially available kit (Innotest PHOSPHO-TAU Antigen, Innogenetics, Belgium). The monoclonal antibodies which are coated on the ELISA plate recognize both the entire moiety and its fragments (Vanmechelen et al., 2000). p-Tau values are expressed as pg/ml.

### Statistical analysis

Clinical and demographic characteristics of the diagnostic subgroups were compared by ANOVA. p-Tau levels in patients, controls and between groups were compared by ANOVA; post-hoc analysis was performed using the Bonferroni adjustment, where appropriate. The specificity and sensitivity were calculated with Graph pad and further confirmed by SPSS 13.0 version where the receiver operating characteristic (ROC) curve; the area under the ROC curve was used as an index of test performance and calculated with a non-parametric method (confidence interval at 95%). The relation between the two variables was calculated using the chi square test. Differences were considered significant if P<0.05.

## Results

### CSF concentration of p-Tau181

**Fig. 1.**
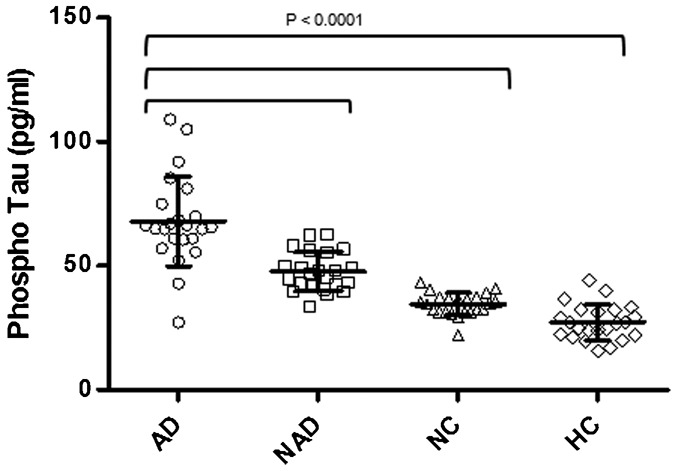
CSF phospho tau in CSF of AD, NAD, NC and HC. AD, 67.87±18.05 pg/ml; NAD, 47.55±7.85 pg/ml; NC, 34.42±4.51 pg/ml; HC, 27.09±7.18 pg/ml; P<0.0001.

The CSF concentrations of p-Tau 181 in AD, NAD, NC and HC are presented in Table 1, which clearly depicts the increase in the concentration of p-Tau 181 in comparison with the NAD, NC and HC. The mean ± SD values for AD was 67.87±18.05 pg/ml which significantly higher than NAD (47.55±7.85 pg/ml), and significantly higher than NC (34.42±4.51 pg/ml) and HC (27.09±7.18 pg/ml) (Fig. 1). The P value of AD with respect to the NAD or NC or HC or NAD and NC and HC or NC and HC is 0.0001.

**Table 1.**
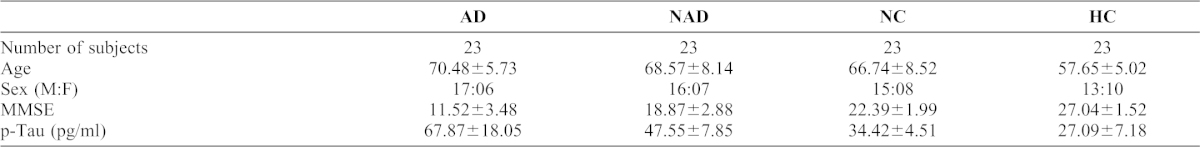
Demographic profile.

### Specificity and sensitivity of AD with respect to the control groups

**Fig. 2.**
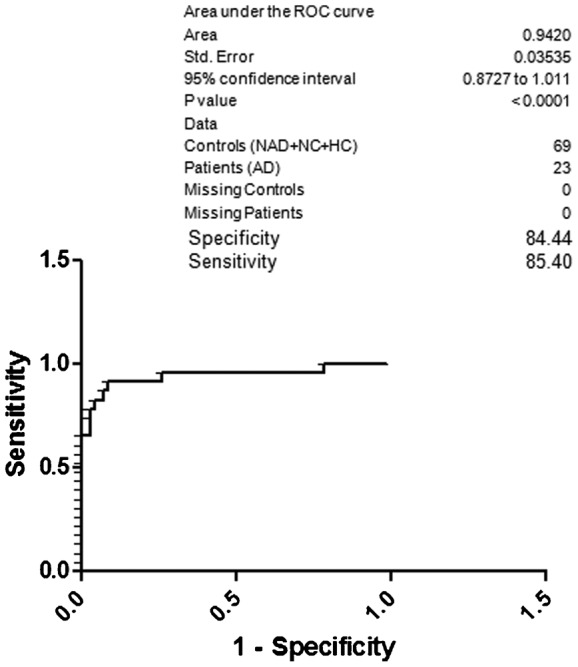
ROC curve; specificity and sensitivity of AD with respect to the NAD, NC and HC.

**Fig. 3.**
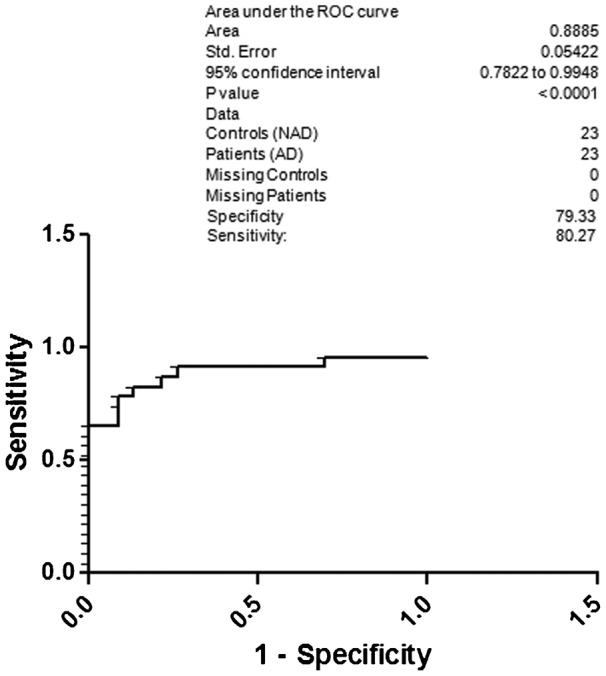
ROC curve; specificity and sensitivity of AD with respect to the NAD.

Table 2 depicts the specificity and sensitivity of AD with respect to the NAD, NC and HC; AD with respect to the NAD; AD with respect to the NAD and NC; AD with respect to the NC; AD with respect to the NC and HC; AD with respect to the HC. Fig. 2 illustrates ROC curve for AD and controls (NAD, NC and HC). The 95% CI was 0.8727 to 1.011. Dichotomous variables were given to the AD and controls in this aspect for calculating the sensitivity and specificity. The specificity and sensitivity of AD with respect to the NAD, NC and HC was 84.44 and 85.40, respectively. Based on the above specificity and sensitivity of AD with three groups, we were curious to find out the specificity and sensitivity of AD with respect to the NAD only because overlapping symptoms and diagnosis difficulty. Fig. 3 depicts area under curve of AD and NAD. Dichotomous variables were given to the AD and NAD for calculating the sensitivity and specificity. The resultant specificity was 79.33, whereas sensitivity was 80.27. The 95% CI in this scenario was 0.7822 to 0.9948.

**Table 2.**
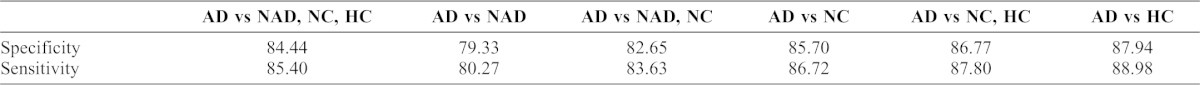
Specificity and sensitivity of AD.

**Fig. 4.**
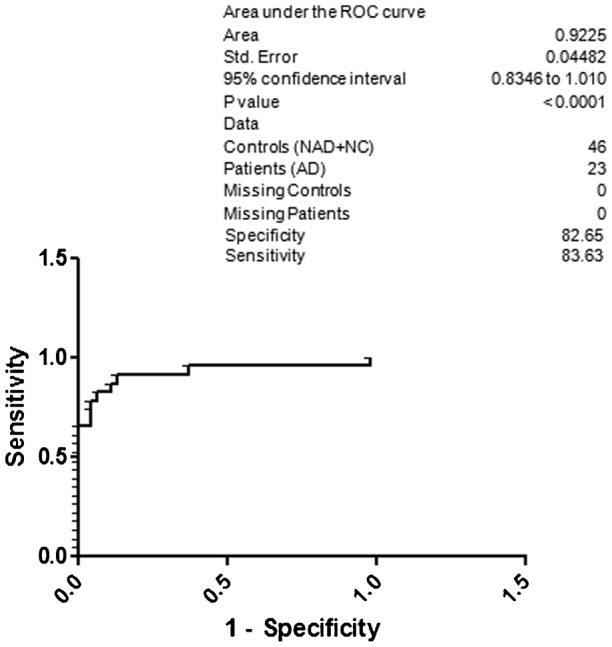
ROC curve; specificity and sensitivity of AD with respect to the NAD and NC.

**Fig. 5.**
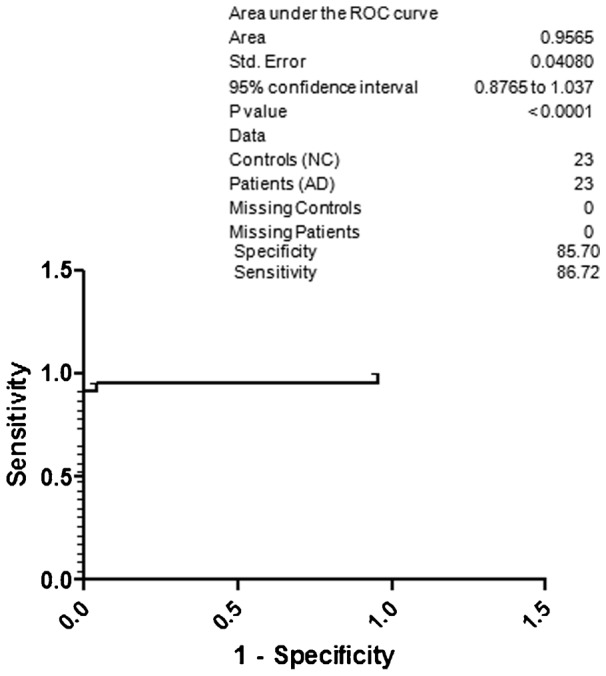
ROC curve; specificity and sensitivity of AD with respect to the NC.

**Fig. 6.**
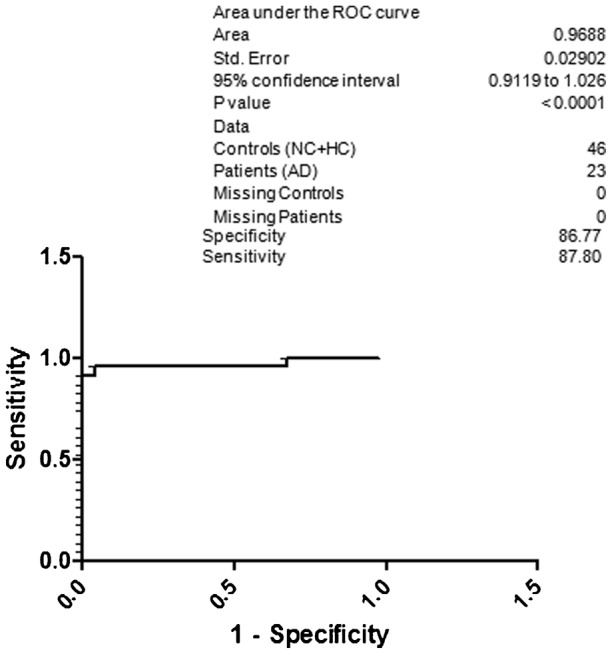
ROC curve; specificity and sensitivity of AD with respect to NC and HC.

**Fig. 7.**
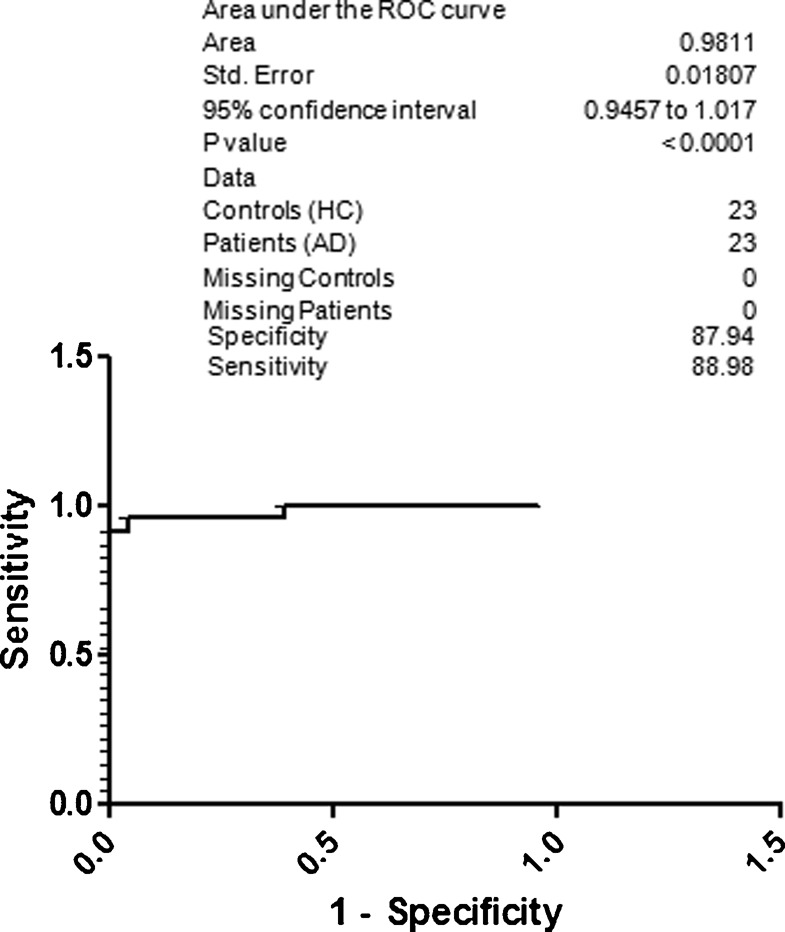
ROC curve; specificity and sensitivity of AD with respect to HC.

For better understanding regarding specificity and sensitivity for the diagnosis of AD based on the CSF p-Tau 181, NAD and NC were included under the control group (Fig. 4). The obtained specificity was 82.65 and sensitivity was 83.63. Surprisingly, the specificity was 85.70 and sensitivity was 86.72 for AD with respect to the NC (Fig. 5). When HC was included along with the NC, the resultant specificity and sensitivity was 86.77 and 87.80, respectively (Fig. 6). The specificity and sensitivity of AD with respect to the HC was 87.94 and 88.98, respectively (Fig. 7).

Table 2 depicts the specificity and sensitivity for the diagnosis AD based on the CSF p-Tau 181 levels with respect to the NAD, NC and HC. Keeping in the mind the overlapping tau pathology and clinical symptoms and MMSE score in NAD and NC patients; the specificity and sensitivity of AD with respect to NAD or NAD and NC could be considerable.

## Discussion and review of literature

In the AD brain hyperphosphorylation of tau causes the formation of NFTs. After analyzing large data, Andreasen in 2003 stated that CSF measurement of Aβ42, t-Tau, and p-Tau not only increases accuracy of clinical diagnosis of AD (Andreasen et al., 2003), but also reliably predicts the conversion to dementia in patients with MCI (Lanari and Parnetti, 2009).

Previous studies have shown that p-Tau levels are increased in AD patients, but there is still uncertainty regarding its qualification as a biomarker from diagnostic point of view. In this study a high sensitivity and specificity for CSF p-Tau levels was obtained by comparing their levels in healthy subjects or non-demented neurological patients. In our study, from North Indian population we found significant increase in CSF p-Tau levels in AD compared with NAD or other subjects. A better sensitivity (80.27) and specificity (79.33) was obtained, when AD compared was with NAD. Further, when AD was compared with NAD and NC we got 82.65 specificity and 83.63 sensitivity. When we compared AD with HC (Fig. 7) the obtained results are in agreement with results published previously which further confirm the important role of phosphorylated species of tau proteins in the diagnosis of AD (Parnetti et al., 2001; Sjögren et al., 2001; Sjögren et al., 2002; Hampel et al., 2004; Maddalena et al., 2003; Riemenschneider et al., 2003).

Tau is a microtubule associated protein and found in both neuronal and non-neuronal cells (Rosso et al., 2003). Various experimental studies highlighted the function of tau in growth, stabilization and assembly of microtubules. Here, the concrete hypothesis suggested that phosphorylation events change the conformational status of tau molecules and leads to the decreased microtubule binding in turn leads to the instability of microtubules because of loss of microtubule assembly (Ishiguro et al., 1999). Although increase in the CSF total tau concentration indicates the damage of neural cells whereas hyperphosphorylation of this tau and accumulation of hyperphosphorylated tau in the NFTs represents the disturbances in the AD brain pathologic process (Brunden et al., 2009; Matsuo et al., 1994). Around 30 phosphorylation sites are present on tau. Hyperphosphorylation of tau occurs either on Ser–Pro or Thr–Pro (present outside microtubule binding domains) during neuronal development and in AD and other neurodegenerative disorders (Blennow et al., 2010; Itoh et al., 2001). The CSF level of p-Tau probably reflects the phosphorylation state of tau. Despite a very marked increase in t-Tau but CSF p-Tau levels are mildly increased in CJD. These data suggest that p-Tau in CSF is not simply a marker for neuronal degeneration or damage but it also specifically reflects the phosphorylation state of tau and thus possibly the formation of tangles in AD brain. Itoh et al. reported a significant increase of p-Tau199 in patients with AD compared with all other non-AD groups (Itoh et al., 2001; Kohnken et al., 2000). In this multicenter study both sensitivity and specificity of CSF p-Tau199 for AD from other studied groups was 85%. Tau phosphorylated at threonine 231 (p-Tau231) seems to be helpful in the differentiation of AD from FTD, vascular dementia (VD), and LBD (Buerger et al., 2002). A follow-up study revealed increased CSF concentration of p-Tau231 at the onset of the disease, followed by decreasing concentrations of p-Tau231 but not total tau, in a group of untreated AD patients. This might suggest a possible role of this isoform in tracking a natural course of the disease (Blennow et al., 1995) Interestingly, tau protein phosphorylated at both positions threonine 231 and serine 235 turned out to be increased in patients with mild cognitive impairment who developed AD during follow up (Hu et al., 2002). In this study a simultaneous evaluation of total tau and phosphorylated tau distinguished the group of patients at risk of developing AD from those who complained of having memory impairment but did not have objective memory loss. Hampel et al. showed equal accuracy of p-Tau181 and p-Tau231, and a slightly worse performance of p-Tau199 when they were simultaneously tested as biomarkers of degenerative disorders (Hampel et al., 2004).

Numerous papers (Table 3 and corresponding references) based on the performance of the different ELISA methods for p-Tau in CSF in the diagnosis of AD (around 1420 AD patients and 708 control got mean sensitivity to discriminate AD from non-demented aged individuals has been 81% and of 91%. Some papers, there has been a relatively large variation in sensitivity and specificity figures of p-Tau between studies using ELISA methods specific for different phosphorylated tau epitopes. Therefore, a study was performed to directly compare the diagnostic performance of p-Tau181, p-Tau199, and p-Tau231 in the same patient material. Among cases with AD, DLB, FTD, VAD, and a group with other neurological disorders, all three p-Tau assays performed equally well in the discrimination of AD from other disorders and non-demented controls. Minor differences found between the assays were that group separation was maximized between AD and FTD using p-Tau231 and between AD and DLB using p-Tau181. Thus, the sensitivity for AD seems equal, whereas minor differences in the phosphorylation of specific tau epitopes between dementia disorders may be reflected in the CSF level of the corresponding p-Tau variant. p-Tau levels in CSF correlate with cognitive decline in patients with mild cognitive impairment (MCI) and with neocortical NFT-pathology in AD (Ravaglia et al., 2008; Mulder et al., 2010). In addition to, both t-Tau and p-Tau predict rate of cognitive decline in different stages of AD (Parnetti et al., 2012; Buerger et al., 2005; Sämgård et al., 2010). Tau is normally phosphorylated at multiple serine and threonine residues, and tau hyperphosphorylation reduces microtubule binding and may enhance aggregation (Brunden et al., 2009). Therefore, it is possible that changes in protein kinases and/or phosphatases could enhance tau phosphorylation. A number of kinases (glycogen syntase kinase 3 (GSK-3), Cyclin-dependent kinase 5 (CDK-5) and microtubule-affinity regulating kinase (MARK) and phosphatases i.e. protein phosphatase 2A (PP-2A) have been implicated as contributing to tau hyperphosphorylation (Matsuo et al., 1994). Currently it is hypothesized that phosphorylation of tau to be a downstream product of Aβ toxicity, and an increase in p-Tau or t-Tau levels in CSF and it is thought to neuronal cell death (Matsuo et al., 1994).

**Table 3.**
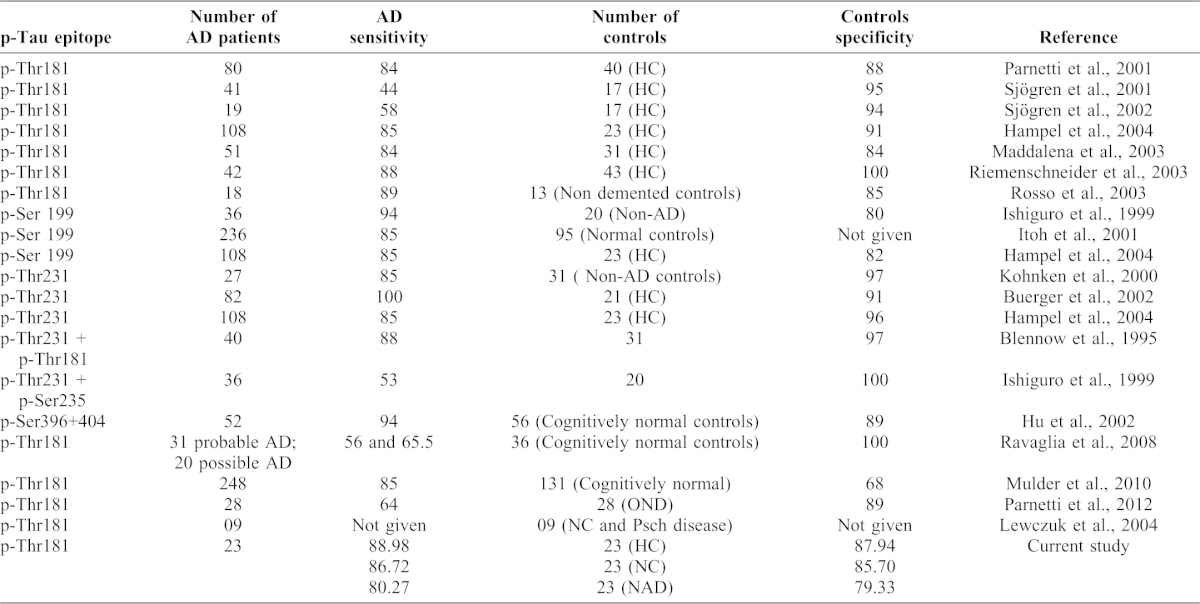
Specificity and sensitivity of AD from previous studies.

## Conclusion

Because of the direct contact of CSF with brain than blood, whatever changes occur during pathology of AD progression they will be directly reflected in CSF. It is noteworthy to mention that tau protein is the first protein which will be released into the CSF so measuring CSF p-Tau could be initial screening marker for the detection of AD from other non-dementing neurodegenerative disorders.
